# Human displacements, fatalities, and economic damages linked to remotely observed floods

**DOI:** 10.1038/s41597-023-02376-9

**Published:** 2023-07-22

**Authors:** Benedikt Mester, Katja Frieler, Jacob Schewe

**Affiliations:** 1 Potsdam Institute for Climate Impact Research, Potsdam, Germany; 2 Institute of Environmental Science and Geography, University of Potsdam, Potsdam, Germany

**Keywords:** Natural hazards, Hydrology, Climate-change impacts, Climate-change impacts

## Abstract

We present a new open source dataset FLODIS that links estimates of flood-induced human displacements, fatalities, and economic damages to flooded areas observed through remote sensing. The dataset connects displacement data from the Internal Displacement Monitoring Centre (IDMC), as well as data on fatalities and damages from the Emergency Events Database (EM-DAT), with the Global Flood Database (GFD), a satellite-based inventory of historic flood footprints. It thereby provides a spatially explicit estimate of the flood hazard underlying each individual disaster event. FLODIS contains two datasets with event-specific information for 335 human displacement events and 695 mortality/damage events that occurred around the world between 2000 and 2018. Additionally, we provide estimates of affected population, GDP, and critical infrastructure, as well as socio-economic indicators; and we provide geocoding for displacement events ascribed to other types of disasters, such as tropical cyclones, so that they may be linked to corresponding hazard estimates in future work. FLODIS facilitates integrated flood risk analysis, allowing, for example, for detailed assessments of local flood-damage and displacement vulnerability.

## Background & Summary

Disaster databases record hundreds of flood events every year and gather information on their societal impacts, such as displacements (recorded by the Internal Displacement Monitoring Centre, IDMC), fatalities and damages (recorded in the Emergency Events Database, EM-DAT). However, they do not include any estimates of the corresponding physical hazard, e.g. inundated area or flood depth. That also means that the exposure to flooding, in terms of affected people and GDP, associated with each disaster is generally unknown.

The delineation of flood hazard, and exposure, in space and time, is however a critical issue for risk analysis^1^. Risk is commonly understood as the product of hazard, exposure, and vulnerability^2,3^. Hazard describes the physical properties of flooding, with people and assets located in floodplains are considered to be exposed. The susceptibility or lack of resilience against the adverse effects of flooding, such as displacement or property damage, is referred to as vulnerability. Flood impacts reported in disaster databases can serve as empirical realizations of risk, and high-resolution estimates of the distribution of population (and partly also GDP/assets) are available for the calculation of exposure. But to understand flood vulnerability, or changes in risk over time, the corresponding hazard must be known. Past studies often used simulated hazard information, e.g., based on modeled annual maximum daily flood water depth^4,5^ or modeled 100 year return period inundation maps^1^. This has many advantages such as extensive geographical and temporal coverage, but the fidelity of the models also puts limitations on the accuracy of flood simulations. Using a single maximum flood value per year can also lead to an underestimation in exposure if more than one flood actually occurred in a given year. Additionally, yearly numbers of affected people and fatalities/damages were used, all neglecting that vulnerability is event-specific^6^.

It is therefore desirable to make use of satellite-based observations of flooded areas, to overcome the limitations of flood model simulations. Satellite-based remote sensors can capture actual flooded areas, implicitly accounting for flood protection^7^ and other non-climate related determinants of flood extent such as land use and infrastructure^8^. Until recently, only a limited number of satellite observations of past floods were publicly available, mainly provided by the archive of the Dartmouth Flood Observatory (DFO; https://floodobservatory.colorado.edu/), and the United Nations Operational Satellite Applications Programme (UNOSAT; http://floods.unosat.org). With the release of the Global Flood Database (GFD; http://global-flood-database.cloudtostreet.info/), a product based on the DFO, an unprecedented inventory of satellite imagery is now available, including 913 large flood events from 2000 to 2018^8^. Here, we connect this new data set of observed flood extents to reported flood impacts, enabling large-N global studies of flood vulnerability, its socio-economic drivers, and related questions. We link impact records from IDMC and EM-DAT with the GFD satellite imagery of historic floods event by event, to develop the new open source dataset FLODIS which provides observed, spatially explicit flood hazard data for hundreds of human displacement and emergency events.

**Fig. 1:**
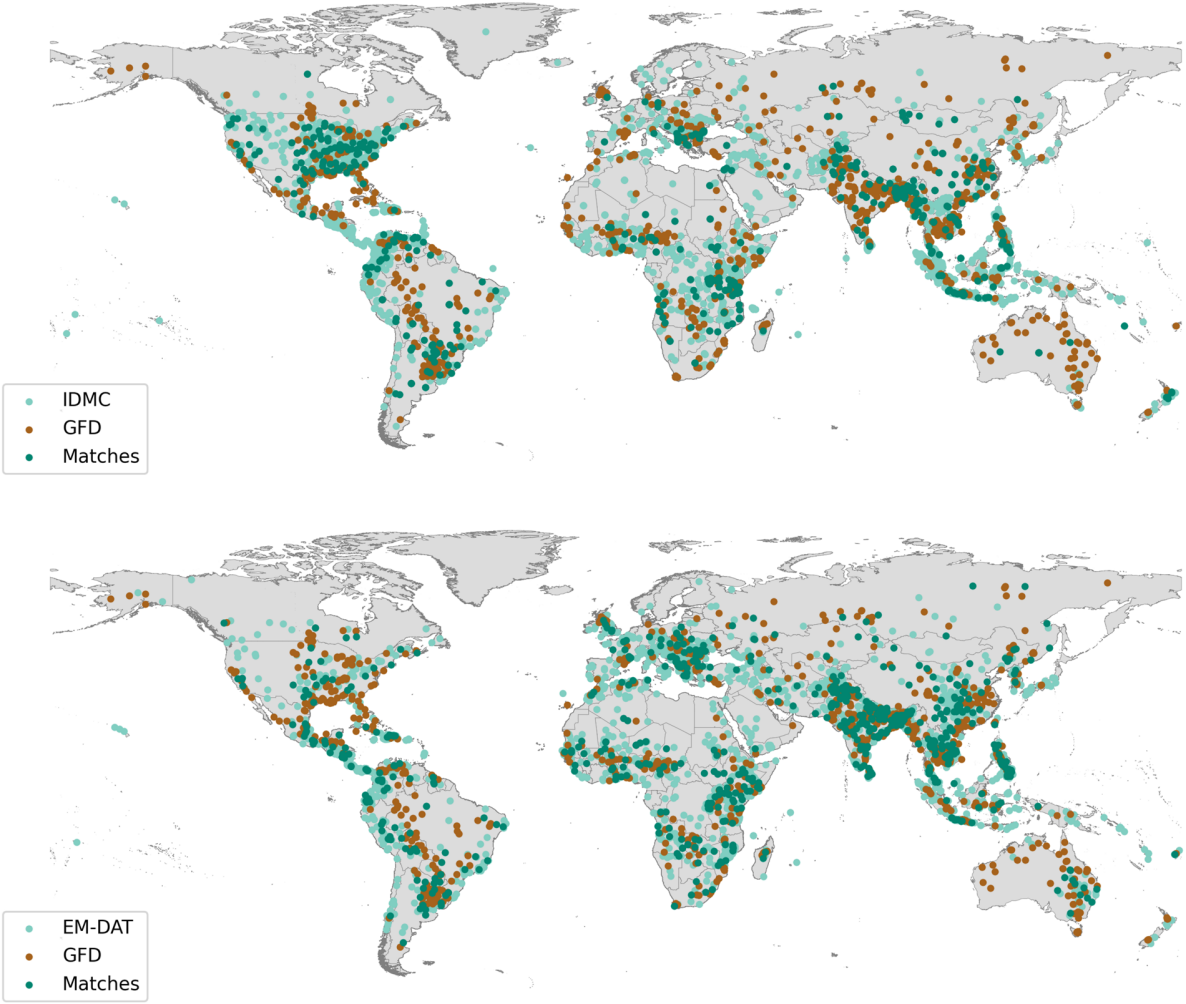
Location overview of all GFD floods (brown), as well as IDMC events (top), and EM-DAT events (bottom) associated with flooding between 2000 and 2018. Affected disaster areas are displayed (light green), as well as the ones successfully matched with the GFD (dark green). Country borders are derived from GADM^10^. Note: Only the centroids of the polygons are shown, some polygons are merged to a single FLODIS disaster entry in the final processing steps.

We first assign an affected area to each entry in the IDMC database, using geographic information contained in the database and a tailored geocoding algorithm. Affected areas for EM-DAT are provided by the Geocoded Disasters (GDIS) dataset^9^. We then match affected areas of IDMC and EM-DAT with the GFD satellite imagery in space and time. The final two FLODIS products contain 335 human displacement (IDMC) entries and 695 mortality/damage (EM-DAT) entries, respectively, all linked with GFD flood extent (Fig. 1). FLODIS allows investigations on cross-country but also within-country variations in flood risk. In addition, we overlay the flood extent with sub-national, rasterized socio-economic data, to estimate the number of affected people, affected GDP, and critical infrastructure as well as event-specific societal and environmental conditions. For example, the 4,255 displacements of the 2016 Louisiana floods (IDMC) are matched in space and time with one of the GFD floods. Intersecting the GFD flood extent with the state border of Louisiana and overlaying it with the gridded data yields, for example, an estimate of 97,916 people affected, 70 hospitals affected, and 7035 km of primary roads affected. The area affected by the floodwaters is characterized by an HDI of 0.80, an urbanization rate of 1.3%, and a percentage of the population over 65 years of age of 21.2%. These and the other affected entities, and socio-economic indicators form a FLODIS displacement entry. The information provided by FLODIS can be used in various ways, e.g., investigating if there is a connection between flood extent (affected entities) and disaster impact. It is also possible to estimate the impact or magnitude of a flood even if it is not listed in a disaster database or GFD, respectively, by comparing it with the several hundred FLODIS entries. Vulnerability to damages or displacement, or mortality may be compared between locations to identify impact hotspots. FLODIS information can also be used to check the plausibility of disaster entries, e.g., by controlling for unusual high/low vulnerabilities. The socio-economic indicators associated with each flood provide assistance in assessing the circumstances in which the event occurred. The extensive set of indicators enable various types of studies, for example multivariate analyses of the drivers of flood impacts or vulnerability. Eight matched IDMC entries and 114 matched EM-DAT entries provide no information on the number of displacements, and fatalities/damages, respectively. FLODIS may help to fill these gaps by determining the exposure and estimating the number of displacements, fatalities, or damages using multivariate analysis. Alternatively, users may extract the matched GFD IDs to retrieve the corresponding GFD flood extent, and perform additional analysis.

## Methods

**Fig. 2:**
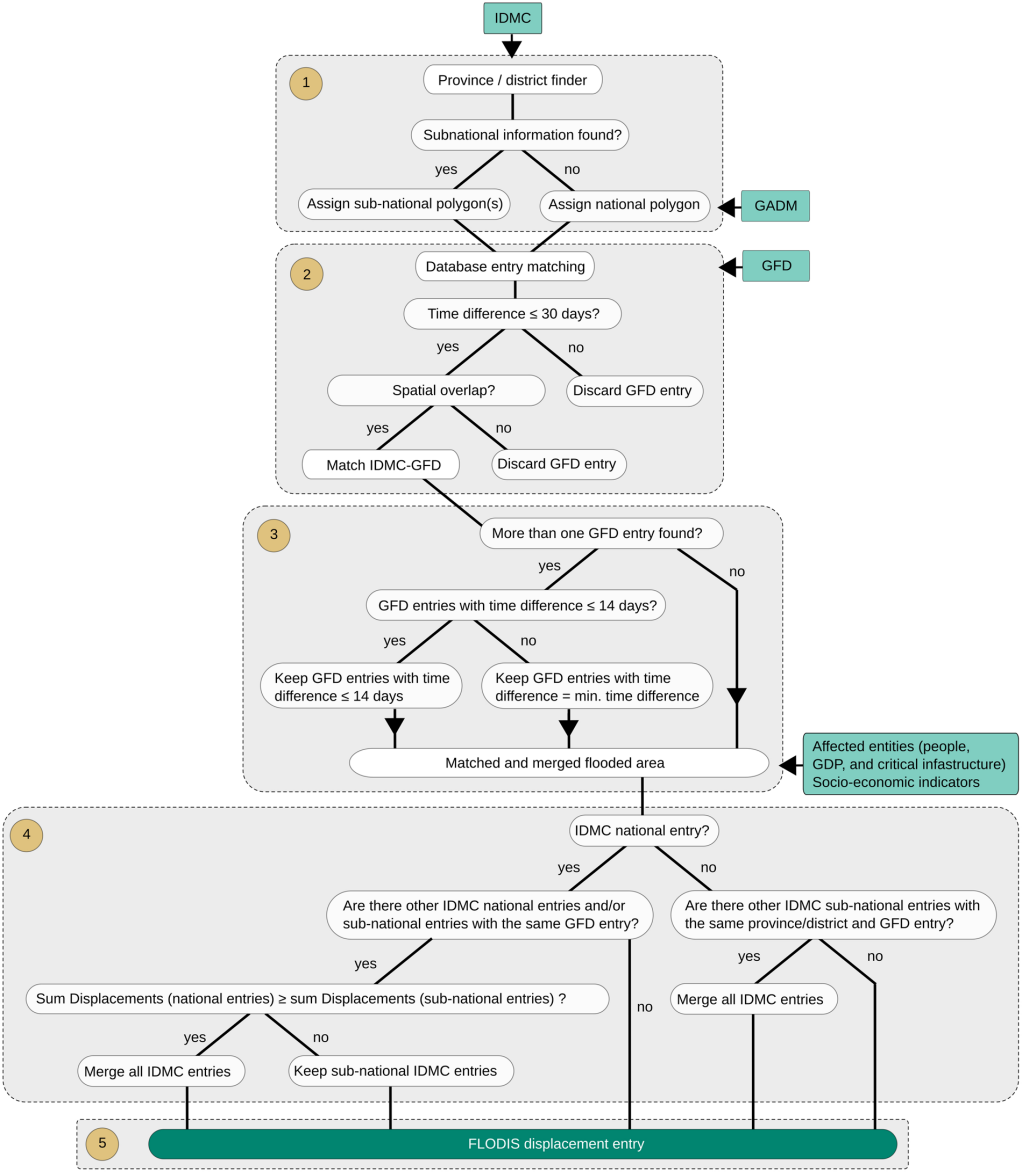
Flowchart of the database merging process between IDMC and GFD, including the used datasets (light green) and processing steps (brown, 1–5). Sub-national information (provinces and districts) is extracted for 3083 entries of the IDMC displacement data bank (**1**). 913 flood events of the GFD serve as a hazard approximation, and are matched with every IDMC entry using location information and time stamps (**2**). If several GFD entries are matched, a multi-criteria scheme is applied to identify and merge flood extents belonging to the same displacement event (**3**). The matched and merged flooded area is used to estimate the affected people, GDP, critical infrastructure as well as sub-national, socio-economic indicators of the disaster. Next, it is checked whether multiple (sub-)national IDMC entries belong to the same disaster (**4**) to generate a final FLODIS displacement entry (**5**).

The IDMC database contains 3083 unique displacement events with the cause being indicated as “Flood” between 2008 and 2018, each including the number of displacement flows and a starting date. For some events, more detailed information is given in the “Event Name” variable. This string can vary from being empty to containing additional information describing the event, such as flood type, duration and affected locations. In a first step we exploit the “Event Name” variable to identify one or several sub-national regions (provinces or districts) where the event occurred (Fig. 2, step 1), using the Global Administrative Areas (GADM) database v.3.4^10^. Note that for provinces and districts in Great Britain we used the 2nd and 3rd sub-national level GADM data, as England, Wales, Scotland and Northern Ireland are listed on the 1st level.

We perform a transliteration process, which converts diacritics to letters of the alphabet for Modern English, e.g., the French accents la cédille “ç” and l’accent aigu “é” become “c” and “e”, respectively. An automated script then calculates for every word within the “Event Name” string its spelling similarity with the 3610 provinces and 45.962 districts in the GADM database, using a similarity score defined as:

1 $$Similarity\;Score=\frac{2\ast M}{T}$$

where M is the sum of sizes of all matched sequences and T is the total number of characters in both sequences. It ranges from 0 to 1, where 1 represents a perfect similarity. A successful match between a IDMC word and a GADM province or district name requires the same ISO3 country code as well as a Similarity Score greater than 0.8. This threshold was found to assure a high degree of similarity while allowing for smaller deviations. For example, the Algerian province “Tamanghasset” (GADM) is written as “Tamanrasset” in an IDMC entry, resulting in a similarity value of 0.87 with M = 10 and T = 23. We additionally computed similarity scores for the “VARNAME_1” and “VARNAME_2” variables in the GADM database, which cover spelling variants of the provinces and districts, respectively, and assigned the province or district to the event if at least one of the scores exceeded 0.8. Referring to the previous example, “Tamanrasset” is also found as a spelling variant in the “VARNAME_1” variable. The geometries of all matched GADM provinces or districts are then merged for every event into a single area, which represents the smallest identifiable area associated with the displacement event (“displacement region”). If no provinces or districts are found, the national area of the country within the event occurred is used. For entries between 2008 and 2012 in the IDMC database, the “Event Name” column is empty, thus the corresponding national areas are used for all those events. Matching displacement events to observed floods, as described below, yields accurate location information for many of these early events too.

For a total of 3083 IDMC entries labeled as “Flood” we geocoded 1702 entries (1157 entries with at least one province, 1074 entries with at least one district, and 529 entries with both at least one province and one district). It should be noted that 688 entries do not contain any event information and others only non-location related information, e.g., “March Floods”.

**Fig. 3:**
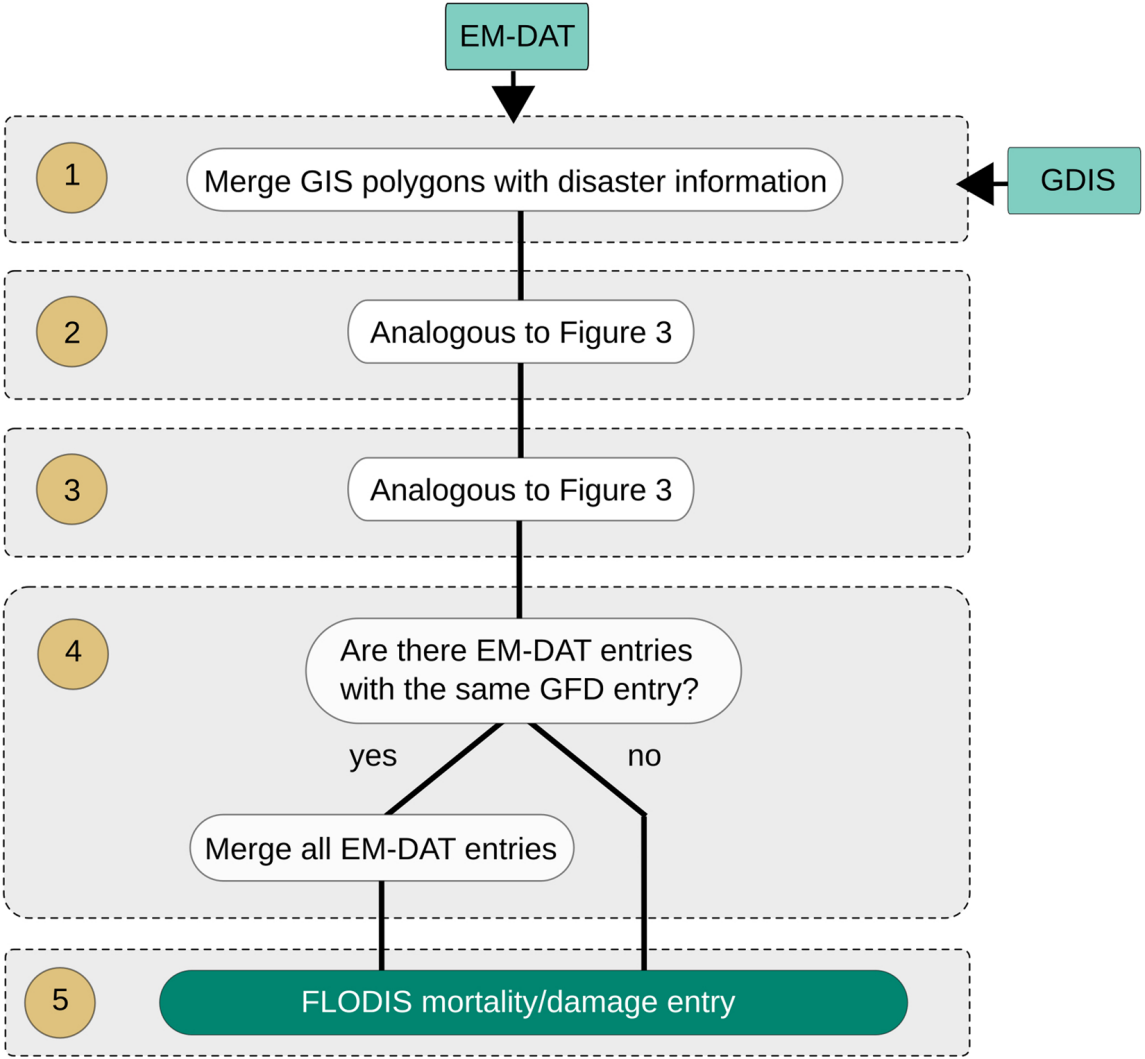
Flowchart of the database merging process between EM-DAT and GFD, including the used datasets (light green) and processing steps (brown, 1–5). EM-DAT disaster entries are merged with the GDIS geographical data using the disaster ID (**1**). Step (**2**) and step (**3**) are analogous to Fig. 2. Next, it is checked whether multiple EM-DAT entries belong to the same disaster (**4**) to generate a final FLODIS mortality/damage entry (**5**).

For information on fatalities and damages, we use the GDIS^9^, which provides GIS polygons for each disaster location in the EM-DAT database (Fig. 3, step 1). We extract 2390 entries with the disaster type “flood” between 2000 and 2018, for which we also obtain the corresponding disaster information, e.g., number of fatalities or total damage, from the EM-DAT database.

In the next step, we perform a spatial and temporal event matching procedure between the IDMC displacement events and EM-DAT/GDIS disaster events on the one hand, and the GFD floods on the other hand (Figs. 2, 3, step 2). The GFD provides surface water extent for 913 large flood events documented by DFO^8^. The data covers the years from 2000 to 2018 and is based on MODIS satellite imagery at 250 m resolution. The matching procedure needs to consider the fact that some flood events affected multiple countries, and could therefore be related to multiple displacement or disaster entries, but are only assigned to one country in the GFD inventory. Simply matching the ISO3 country codes between GFD and the IDMC or EM-DAT databases is therefore not sufficient, and we instead consider the actual spatial overlap between flooded area and the region associated with the disaster, as identified above. A second consideration is that distinct floods may have occurred simultaneously in the same country but in different locations, and we use both temporal and spatial information to avoid false matches in such cases.

**Fig. 4:**
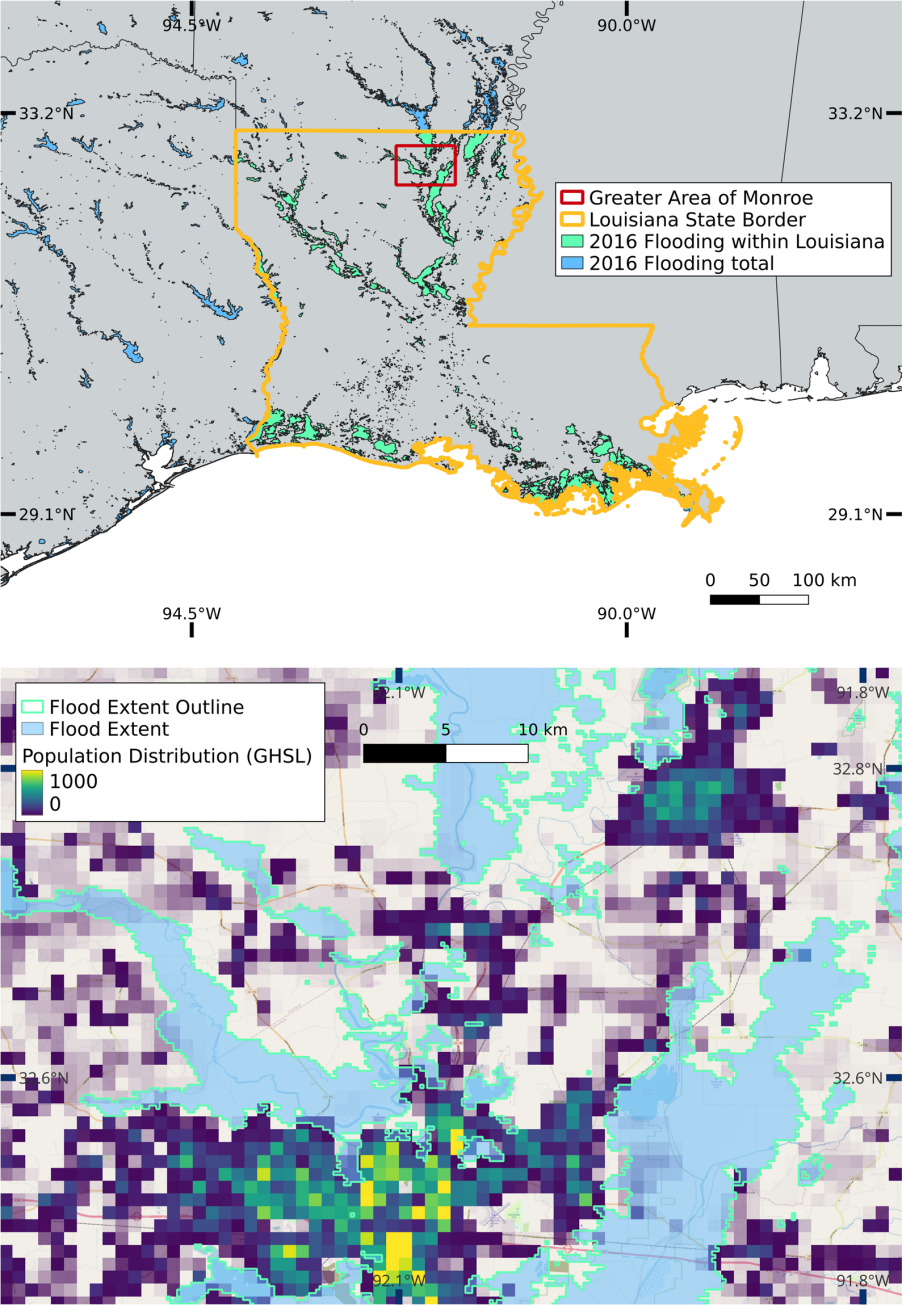
The 2016 Louisiana floods (blue) are shown in the upper panel, including overlapping flood extent (cyan) with the state of Louisiana (orange) and the outline for the greater area of Monroe (red rectangle). Country and state borders are derived from GADM^10^. GHSL population distribution^11^ is overlaid with the flood extent for the greater area of Monroe (lower panel), background is retrieved from © OpenStreetMap^24^.

Two conditions must be hence fulfilled for a successful match: (i) the affected disaster area must intersect with the GFD event polygon and (ii) the starting date difference must be equal or less than 30 days. We thereby ensure a merging of the databases with high accuracy while allowing for minor deviations in time and space due to, e.g., inaccurate or ambiguous starting date information (e.g. the displacement database might indicate the first day of the month when the exact day is unknown; or the date of satellite observation might not match the date of the disaster record during longer events). For example, for the 2016 Louisiana Floods the IDMC database lists an entry with the Event Name “Louisiana floods (March)” and the date “2016-03-08”. Connecting the geocoded affected area (state of Louisiana (province)) with the two matching conditions (i) and (ii) shows a spatial and temporal overlap with the GFD ID 4337 (2016-03-08 to 2016-03-25) (Fig. 4). If two or more GFD entries are matched to a single disaster entry, we keep entries with a starting date difference equal or less than 14 days. In the case no entries meet this criteria, the smallest date difference for this event is determined and GFD entries with a higher date difference are discarded (Figs. 2, 3, step 3). The resulting GFD flooded areas are merged and cropped to the extent of the IDMC/EM-DAT affected disaster area.

We then compute the number of affected people using the Global Human Settlement Layer (GHSL)^11^, and the gridded population of the world version 4 (GPW)^12^, the number of affected GDP using gridded GDP counts^13^ and the number of affected critical infrastructure entities/units. As the population and GDP count data is only available for certain years (GHSL: 2000 and 2015; GPW 2000, 2005, 2010, 2015, and 2020; GDP: 2000–2015), we linearly interpolate/extrapolate the missing years to create a continuous annual time series for every dataset between 2000 and 2018. The critical infrastructure entities are extracted from the Critical Infrastructure (CI) dataset^14^, which is based on Openstreetmap and lists the number of entities per grid cell, e.g., 20 communication masts are located in one cell. The information on critical infrastructure is based on the year 2022 and values within FLODIS are assumed to be the same for the period of 2000 to 2018. By overlaying the (merged) GFD flood extent with each gridded dataset, we compute the sum and mean of affected entities, accounting for every grid cell touched by the flood extent. Additionally, we also append FLODIS with a set of gridded socio-economic indicators^11–13,15–21^ by computing the mean of all grid cells touched by the flood extent. A complete overview of all input datasets and relevant metadata is available on the Zenodo repository^22^. If several disaster entries are assigned to the same GFD flood(s) and the same country, all entries are merged to link the GFD flood extent to one combined disaster (Figs. 2, 3, step 4). This procedure reduces the number of displacement entries from 524 to 335, and the number of mortality/damage entries from 816 to 695. In the last step, the entries are cleaned and merged to the final FLODIS displacement dataset (Fig. 2, step 5) and the final FLODIS mortality/damage dataset (Fig. 3, step 5), respectively. An overview of all FLODIS attributes is presented in Tables 1, 2.

**Table 1:** Overview of FLODIS disaster impact information, affected entity information, and metadata.

Attribute Name	Attribute Type	Unit	Description
displacements*	Impact	—	Number of displacement flows
total_deaths**	Impact	—	Sum of death and missing
no_injured**	Impact	—	Number of injured people
no_affected_EMDAT**	Impact	—	Number of affected people according to EM-DAT
no_homeless**	Impact	—	Number of homeless
total_affected_EMDAT**	Impact	—	Sum of injured, homeless, and affected according to EM-DAT sources
total_damages_(000_USD)**	Impact	US$ (‘000)	The amount of damage to property, crops, and livestock
pop_affected_sum_GHSL	Affected entity	—	Sum of people affected (analogous for GPW)
GDP_affected	Affected entity	PPP const. 2011 int. USD	Sum of GDP affected
{CISI_variable}_sum	Affected entity	{Variable}	Sum CISI variable affected
ISO3	Metadata	—	Country code
year	Metadata	Year	Year of the event
disasterno**	Metadata	—	Disaster number
GID_1*	Metadata	—	List of affected provinces (GADM code)
GID_2*	Metadata	—	List of affected districts (GADM code)
num_provinces*	Metadata	—	Number of affected provinces
num_districts*	Metadata	—	Number of affected districts
GFD_matches	Metadata	—	Number of matches with the GFD
GFD_matches_nr	Metadata	—	List of matched GFD IDs
GFD_matches_time_dif	Metadata	Days	(Mean) time difference in days between disaster entry and GFD flood(s)
matching_type*	Metadata	—	Type of matching, see Fig. 2 step 4

“*” and “**” indicate that the attribute is only available for the displacement dataset and mortality/damage dataset, respectively. “{CISI_variable}” relates to the various critical infrastructure variables (cf. the input data information in the Zenodo repository^22^).

**Table 2:** Overview of all FLODIS (socio-economic) indicators.

Attribute Name	Attribute Type	Unit	Description
GFD_duration	(Socio-economic) Indicator	Days	(Mean) duration time of GFD flood(s)
pop_affected_mean_GHSL	(Socio-economic) Indicator	People/(0.5 arcmin)²	Mean number of people affected per population grid cell (analogous for GPW)
pop_density_GHSL	(Socio-economic) Indicator	People/km²	Population density in the flooded area (analogous for GPW)
GDP_affected_mean	(Socio-economic) Indicator	PPP const. 2011 int. USD/(5 arcmin)²	Mean number of GDP affected per grid cell
{CISI_variable}_mean	(Socio-economic) Indicator	{Variable}/(6 arcmin)²	Mean CISI variable affected per grid cell
GDPpc_mean	(Socio-economic) Indicator	PPP const. 2011 int. USD	Mean GDP per capita
HDI_mean	(Socio-economic) Indicator	—	Mean HDI
urbanization_mean	(Socio-economic) Indicator	% of total area	Mean urbanization
landuse_total_mean	(Socio-economic) Indicator	% of total area	Mean land use
elevation_mean	(Socio-economic) Indicator	—	Mean elevation
roughness_mean	(Socio-economic) Indicator	—	Mean roughness
slope_mean	(Socio-economic) Indicator	—	Mean slope
female_mean	(Socio-economic) Indicator	% of total population	Mean female population
pop_0_14_mean	(Socio-economic) Indicator	% of total population	Mean population ages 0–14 years
pop_65_plus_mean	(Socio-economic) Indicator	% of total population	Mean population ages 65 + years
FLOPROS_merged_mean	(Socio-economic) Indicator	Protection against return period in years	Mean FLOPROS “merged” level
FLOPROS_modeled_mean	(Socio-economic) Indicator	Protection against return period in years	Mean FLOPROS “modeled” level
forest_cover_mean	(Socio-economic) Indicator	% of total area	Mean forest cover

“{CISI_variable}” relates to the various critical infrastructure variables (cf. the input data information in the Zenodo repository^22^).

## Data Records

FLODIS is publicly available from a Zenodo repository^22^, and the following GitHub repository: https://github.com/BenediktMester/FLODIS^23^. Two FLODIS datasets exist: IDMC displacement events linked with GFD entries (“FLODIS displacement”), and EM-DAT fatality and damage events linked with GFD entries (“FLODIS mortality/damage”). Both datasets are available as CSV files, and include the GFD (DFO) IDs, GDIS disaster numbers (for FLODIS mortality/damage), and GADM codes of affected provinces and districts (for FLODIS displacement). No GFD flood extents are provided, however, the GFD (DFO) IDs may be used to look up the corresponding GIS polygons, and recreate the flood extent data using scripts (see the Code Availability section). The GitHub repository and Zenodo repository contain an additional CSV file providing detailed information on the input datasets used. The repositories also contain a fully geocoded IDMC displacement dataset appended with the detected provinces and districts and the corresponding GADM sub-national codes; this also includes displacement events which were not matched with any flood data as well as other hazard types. For a total of 11,731 IDMC entries we geocoded 7,915 entries (5,893 entries with at least one province, 5,740 entries with at least one district, and 3,718 entries with both at least one province and one district. 1,202 entries do not contain any event information. The data is available as a CSV file.

## Technical Validation

As FLODIS is the first data product of its kind, a direct comparison with existing datasets is not possible. Nonetheless, to assess the quality of FLODIS, we analyze the detection rates of the geocoding algorithm, and check for temporal and spatial biases within the FLODIS datasets.

**Fig. 5:**
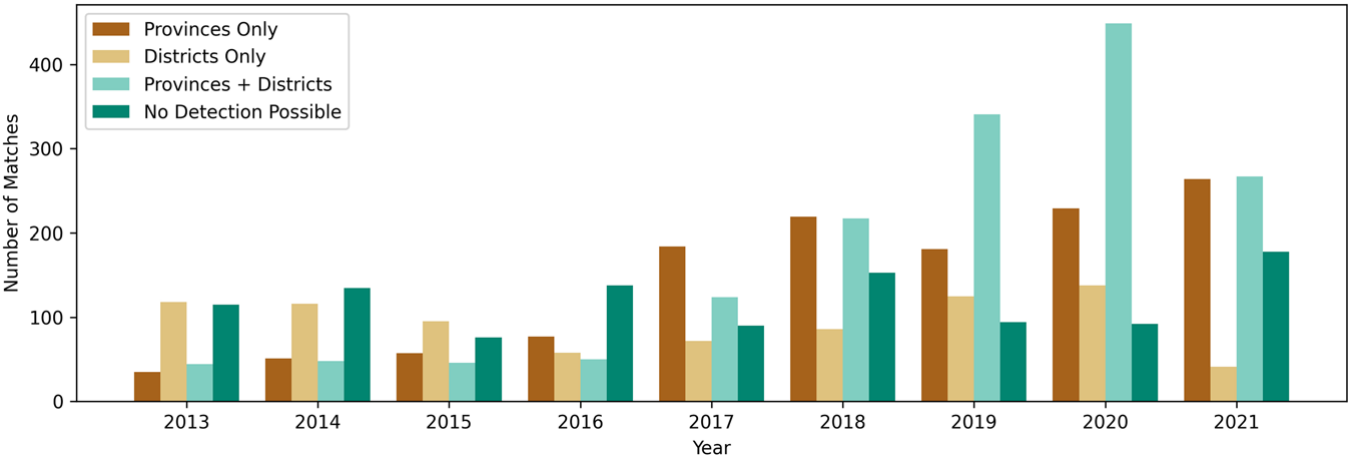
Number of detected provinces and districts by the IDMC database geocoding over time. No subnational event information is available before 2013.

**Fig. 6:**
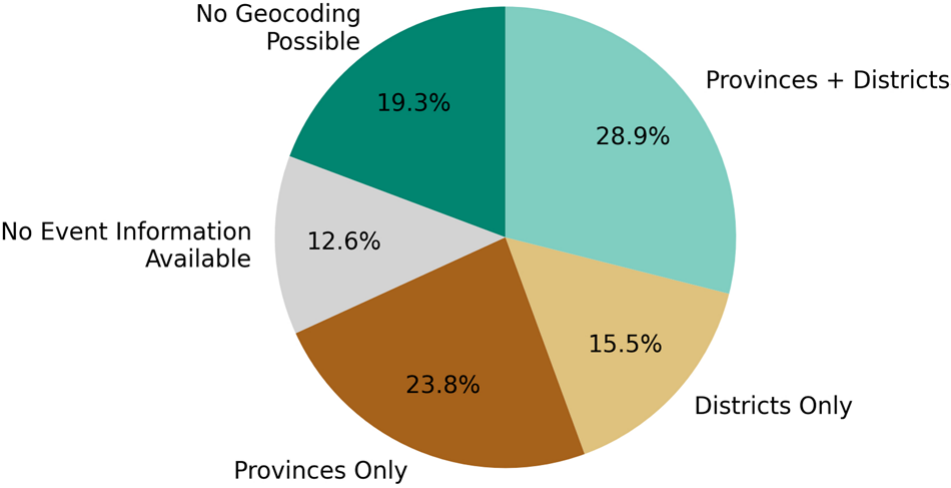
Share of detected provinces and districts by the IDMC database geocoding (2008–2021). Note: prior to 2013 no event information is available.

First, we assess the geocoding algorithm used to extract subnational information out of IDMC entries. The number of entries within the IDMC database continuously increases between 2013 and 2021 (Fig. 5). The share of entries without any detectable subnational information is decreasing, which indicates an improved reporting on affected locations over time. For almost 20% of all IDMC entries no geocoding is possible, even though event information is available (Fig. 6). This can be mostly explained by missing location information, or the geocoding algorithm failing to extract the information, e.g., if it is embedded in more complex sentence structures. The number of detected provinces is higher than the number of detected districts, which likely reflects that detailed subnational disaster information is still often missing.

**Fig. 7:**
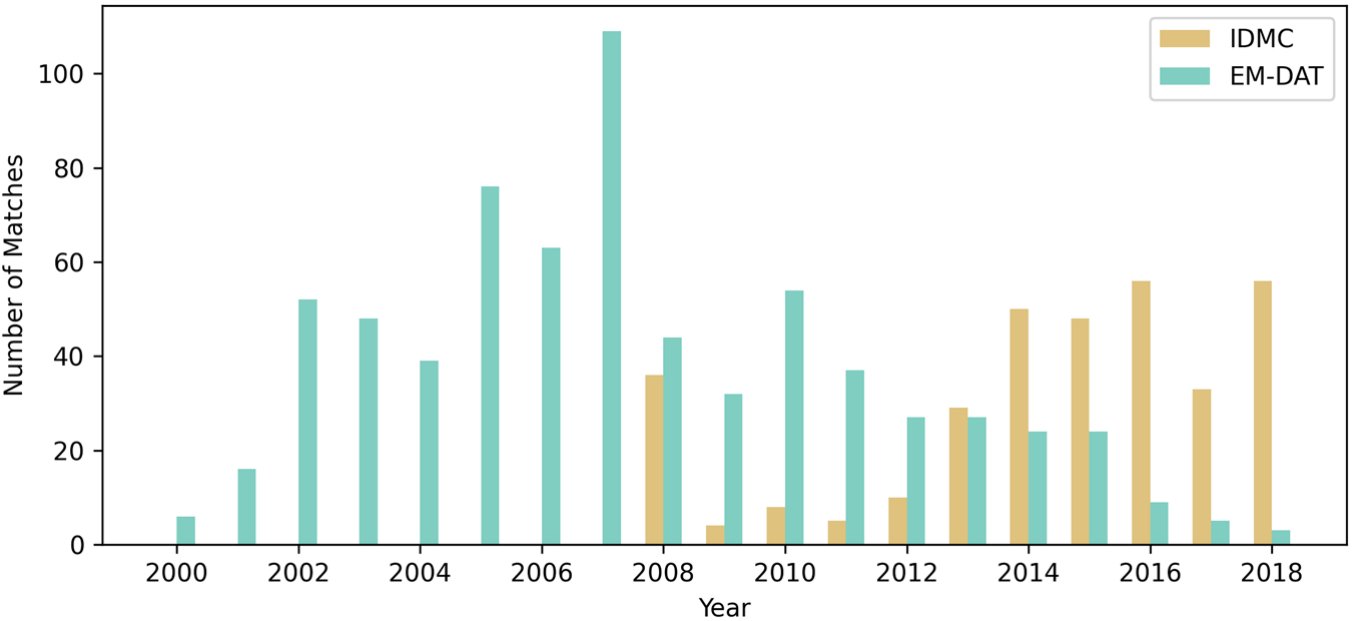
Number of GFD matches with IDMC/EM-DAT per year over time. Note: No subnational event information is available for the IDMC database before 2013.

**Fig. 8:**
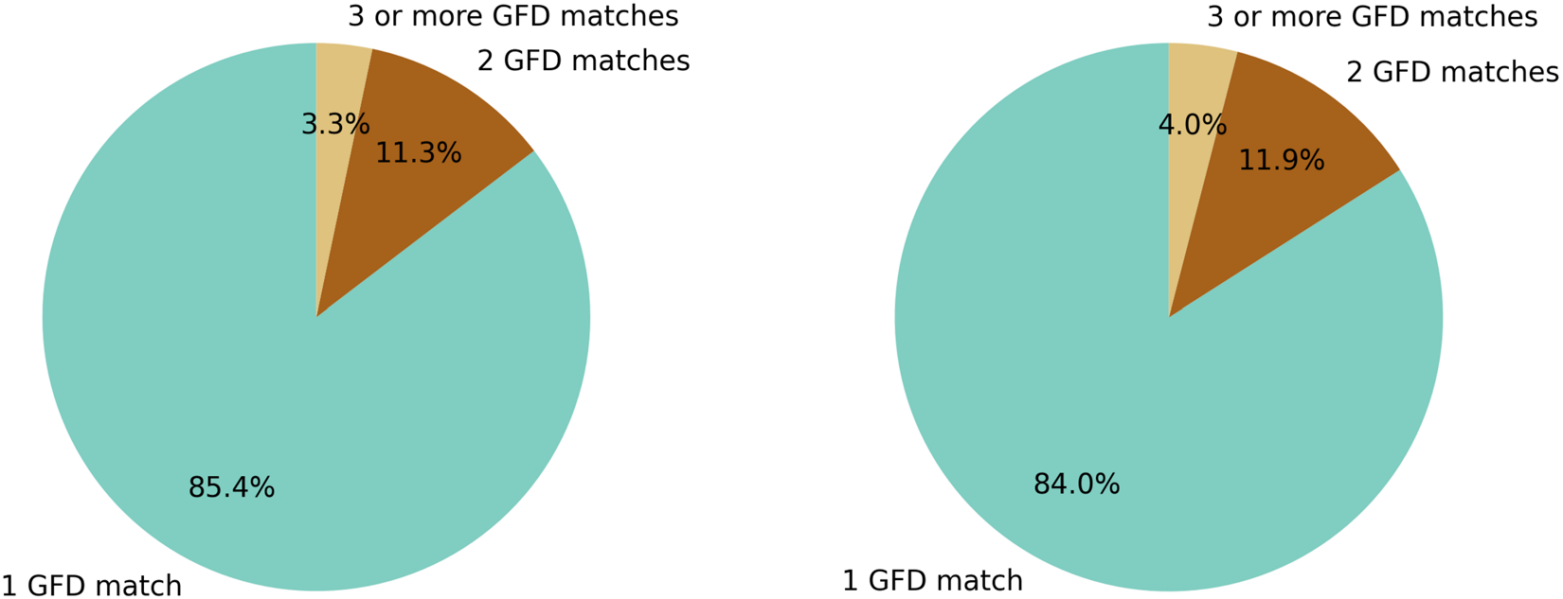
Number of GFD events matched per IDMC entry (left) and EM-DAT entry (right).

We then determine the number of matches between the GFD and IDMC/EM-DAT over time. Starting in 2014, the number of successful matches increases for the IDMC database, which can be mostly explained with an increase in event reporting (Fig. 7). The rate for the EM-DAT quickly increases, peaks in 2007, and then continuously decreases. Global warming, population growth, and shifting settlement patterns lead to changes in event frequency, however, the number of yearly reported EM-DAT entries tends to have stabilized since the early 2000s^9^. Most matches are located in India, China, and USA; for IDMC many matches can be also found in Indonesia and Argentina, whereas matches for EM-DAT accumulate in Pakistan and the Philippines (Fig. 1). It is observable that a low ratio of matched IDMC events is present in some countries and world regions, e.g., Afghanistan or Central America. Additionally, more events are reported by the GFD than by the disaster databases in some regions, e.g., several provinces in Australia. For both databases, approximately 85% of all matches are associated with only one GFD entry, while matches with two (11–12%) or more GFD entries (3–4%) are relatively rare (Fig. 8).

## Usage Notes

FLODIS is based on multiple data sources which differ, for example, in terms of geographical coverage, under-reporting, source, or terminology. IDMC employs event-based monitoring to track displacement numbers without any threshold in terms of the number of people displaced (https://www.internal-displacement.org/monitoring-tools). The estimates are based on homes destroyed or uninhabitable, households or families displaced, and mandatory evacuations. Events enter EM-DAT if ten or more people are reported killed, or 100 or more people are reported affected. Additionally, the declaration of a state of emergency or the call for international assistance can also be a criterion (https://www.emdat.be/explanatory-notes). DFO, which is the basis of GFD, considers “large” flood events, which are characterized by, for example, substantial damage, high reported return period, or fatalities (https://floodobservatory.colorado.edu/Archives/ArchiveNotes.html). These different catalog criteria may explain unmatched events as well as the partial difference between the FLODIS displacement and FLODIS mortality/damage datasets; as such both data products should be viewed as complementary rather than congruent. In addition to information on the “No Injured”, “No Affected”, and “Total Affected”, the FLODIS mortality/damage dataset also provides estimates on the”No Homeless”, which are based on the number of destroyed/heavily damaged houses. As different methodologies exist to derive the number of displacements (IDMC) and homeless (EM-DAT), the two numbers cannot be directly compared.

The analysis of FLODIS datasets can be extended by calculating derivatives, e.g., the vulnerability by dividing an observed impact with the number of affected entities. The FLODIS entries can also be appended with additional affected entities or socio-economic indicators using the temporal information, the provided disaster area IDs for gridded datasets, or ISO3 codes for national information. The corresponding scripts are provided on GitHub (see Code availability section). Affected assets can be computed using the affected GDP and annual national data on capital stock from, for example, the Penn World Table (https://www.rug.nl/ggdc/productivity/pwt/).

The geocoded IDMC dataset may be linked with other hazard databases using the provided start date information, GADM codes, and the matching scripts. The geocoding script may also be used to identify locations within other disaster database entries. A manual/automatic inspection is recommended to ensure a sufficiently high geocoding rate. The Similarity Score threshold can be adjusted for this purpose.

## Data Availability

The source code for this study is publicly available through the following GitHub repository: https://github.com/BenediktMester/FLODIS^23^. It includes the geocoding algorithm to extract subnational information out of the IDMC database, as well as the main scripts to match the IDMC/EM-DAT database with the GFD and to produce the two final FLODIS datasets. We also provide code for the preprocessing of input datasets, the validation procedure, and the development of the figures.
